# Genetic and genomic analysis of reproduction traits in holstein cattle using SNP chip data and imputed sequence level genotypes

**DOI:** 10.1186/s12864-024-10782-5

**Published:** 2024-09-19

**Authors:** Leopold Schwarz, Ana-Marija Križanac, Helen Schneider, Clemens Falker-Gieske, Johannes Heise, Zengting Liu, Jörn Bennewitz, Georg Thaller, Jens Tetens

**Affiliations:** 1 Department of Animal Sciences, Georg-August-University, 37077 Göttingen, Germany; 2 Institute of Animal Science, University of Hohenheim, 70599 Stuttgart, Germany; 3 Vereinigte Informationssysteme Tierhaltung w.V. (VIT), 27283 Verden, Germany; 4 Institute of Animal Breeding and Husbandry, Christian-Albrechts-University, 24118 Kiel, Germany

**Keywords:** GWAS, Dairy cattle, Health traits, Fertility, X chromosome

## Abstract

**Background:**

Reproductive performance plays an important role in animal welfare, health and profitability in animal husbandry and breeding. It is well established that there is a negative correlation between performance and reproduction in dairy cattle. This relationship is being increasingly considered in breeding programs. By elucidating the genetic architecture of underlying reproduction traits, it will be possible to make a more detailed contribution to this. Our study followed two approaches to elucidate this area; in a first part, variance components were estimated for 14 different calving and fertility traits, and then genome-wide association studies were performed for 13 reproduction traits on imputed sequence-level genotypes with subsequent enrichment analyses.

**Results:**

Variance components analyses showed a low to moderate heritability (h^2^) for the traits analysed, ranging from 0.014 for endometritis up to 0.271 for stillbirth, indicating variable degrees of variation within the reproduction traits. For genome-wide association studies, we were able to detect genome-wide significant association signals for nine out of 13 analysed traits after Bonferroni correction on chromosome 6, 18 and the X chromosome. In total, we detected over 2700 associated SNPs encircling more than 90 different genes using the imputed whole-genome sequence data. Functional associations were reviewed so far known and potential candidate regions in the proximity of reproduction events were hypothesised.

**Conclusion:**

Our results confirm previous findings of other authors in a comprehensive cohort including 13 different traits at the same time. Additionally, we identified new candidate genes involved in dairy cattle reproduction and made initial suggestions regarding their potential impact, with special regard to the X chromosome as a putative information source for further research. This work can make a contribution to reveal the genetic architecture of reproduction traits in context of trait specific interactions.

**Supplementary Information:**

The online version contains supplementary material available at 10.1186/s12864-024-10782-5.

## Background

Breeding programs in dairy cattle have in the past decades focused on performance, even accelerated by genomic selection. This has resulted in a significant increase in milk production, but has had a negative impact on reproduction traits due to antagonistic genetic relationships [1–3]. Boundary conditions for breeding for higher reproductive performance are the low heritability of reproduction traits, in contrast to the moderate heritability of production traits [4–6]. Genomic selection, however, has been shown to be a useful tool in breeding for low heritability traits, as demonstrated in the German Holstein population [7]. In this context, an increasing consideration and reallocation of the weighted traits included in the total merit index is being applied in practice [7, 8]. Furthermore, decreased fertility has an impact on both, animal welfare and the economics of dairy production [2]. Impaired fertility remains one of the major factors for culling animals, accounting for up to 20% of all culling reasons in dairy cattle [9, 10]. The additional costs of poor fertility are also clearly shown in the estimation of the total merit index (RZ€) for German Holsteins, where, for example, stillbirth is calculated with a cost of €137.50 per case [11]. This highlights the close relationship between animal welfare and economically successful dairy farming, particularly in German Holstein breeding, with regards to reproductive performance. Improving reproductive efficiency can increase animal welfare by reducing the proportion of involuntary culling in herds, lowering individual animal diseases, and simultaneously reducing variable costs for dairy farmers while enhancing their merit.

To gain insights into the regulatory pathways affecting fertility and identify associated genomic regions, genomic studies and the use of molecular genetic markers have been established as effective methods [12, 13]. Several studies have investigated genetic associations for reproduction traits in Holstein dairy cattle using genome-wide association studies (GWAS) on autosomes [14–16]. Capturing the causal region or quantitative trait loci (QTL) of interest depends on both the density of markers used and the structure of linkage disequilibrium (LD) [17]. Therefore, the accuracy is limited due to the distribution of marker arrays used and the LD structure behind them [18]. Beneficial, the usage of whole-genome sequence (WGS) data is decoupled from LD structure dependency since the causative mutation itself is likely included [17]. Moreover, using WGS data in GWAS allows fine mapping for complex traits [19] and offers high potential for revealing the underlying genetic architecture, including quantitative trait nucleotides (QTN) [17, 20, 21]. In addition to sequencing animals, genotype imputation has the potential to predict genotypes that have not been directly genotyped in a study, based on a reference panel of haplotypes [22]. Imputation combines the advantages of high marker density in WGS data with the ability to predict large-scale cohort data in a cost-effective manner, to be used for GWAS or fine-mapping [23].

The aim of this study is to improve the genetic characterisation of reproduction traits in German Holstein cattle and to identify putative influential genomic regions using chip and imputed WGS data. For pedigree-based analyses based on first lactation phenotypic records, we initially included a group of 34,497 primiparous German Holstein cows. In addition, we utilised imputed WGS data for GWAS of 13 reproduction traits using a mixed linear model association (MLMA) [24]. Our approach identified more than 2700 distinct genome-wide significantly associated SNPs and promising genomic regions on autosomes and the X chromosome. Finally, we conducted enrichment analyses to contextualise our findings.

## Material & methods

### Animals and phenotypes

Phenotypic and SNP chip data were provided from the “KuhVision” project, a cow reference population including 252,285 German Holstein cows representing the genomic pattern of German Holstein Friesian [7]. The national computing centre VIT (Vereinigte Informationssysteme Tierhaltung w.V., Verden, Germany) provided all data for animals included [7]. This large dataset was filtered according to the steps described below in order to ensure a sufficient number of observations in each effect class. The intention is thereby a potentially strong environmental impact, that needs to be accounted for in the analyses. At this, having a sufficient number of observations in each class would make sure that the environmental and genetic effects can precisely be estimated and differentiated from each other. After filtering, a subset of 34,497 primiparous Holstein cows born between 2013 and 2018 with observations for reproductive performance and 13 disease traits, including the three reproduction diseases of interest in this study, was available. In general, the disease traits were recorded on farms, mainly by the farmers themselves, claw trimmers, and veterinarians. They were binary coded, which implies that 0 means “no disease during the lactation” and 1 “at least one event of disease during the lactation”. At this, multiple disease events over the course of the lactation were not considered. In order to generate the final dataset of 34,497 cows, we applied the following filter steps. First, all cows with less than 270 and more than 305 days in milk (DIM) were excluded, resulting in a mean of 302.43 DIM in the final dataset. Then, all cows whose age at first calving (AFC), recorded in months, was below 21 and above 36 months were excluded. Thus, the mean AFC in the final dataset was 24.83 months and each class of AFC consisted of at least 20 individuals. Next, our intention was to avoid biased results because of farms with an incomplete recording of disease cases. Hence, farms with less than 10 cows having an event of diseases during their lactations were excluded, resulting in a total of 103 farms having on average 345 cows per farm. In the last step, the multicode herd-year-season (hys) was generated by combining the cow’s farm, year and season of calving, whereby the partitioning of seasons followed the calendric partitioning. Here, the filtering implied that each class of hys had to consist of at least 20 individuals. Finally, the pedigree consisted 90,407 animals covering two complete generations in the final dataset. The workflow and filtering criteria were first described in Schneider et al. [25].

### Phenotypic trait data

A total of 13 fertility and calving traits were considered for which breeding values are routinely estimated (Table 1). The deregressed proofs (DRPs) were based on the official breeding value estimation in 2021. Basic principle for the calculations are based on the deregression method firstly presented by Jairath et al. [26]. The adaption of this method on estimated breeding values in German Holstein cattle is described by Liu and Masuda [27]. For the pedigree-based analyses, we included 34,497 animals for each trait. In contrast, the number of available DRPs per trait ranged from 24,559 (calving ease direct, **CEd**) up to 34,494 (stillbirth maternal, **SBm**) available for genome-wide association analysis (Table 1).

**Table 1:** Overview about phenotypic traits used for GWAS

Abbreviation	Trait	Definition	No.
CEd/CEm	Calving ease direct (d)/maternal (m)	difficulty of calving, recorded in four classes for all cows under milk recording in all parities	24,559/34,494
CFc	Calving to first insemination	time from calving to first insemination in days	34,210
DOc	Days open	timespan from calving to successful insemination, indirect calculated	34,210
FSc/FSh	First to successful insemination cows (c)/heifers (h)	time from first to successful insemination in days	34,103/30,728
MET	Endometritis/Metritis	cases within first hundred days in lactation	27,283
NGV	Retained placenta	observations until day seven after calving are taken into account	28,182
NRc/NRh	Non-return rate 56 cows (c)/heifers (h)	re-insemination registered within 56 days after the first insemination	34,186/30,729
SBd/SBm	Stillbirth direct (d)/maternal (m)	“All-or-None” trait. After calving the calf was born dead or died within 48 h is considered as stillborn	25,998/34,494
ZYS	Ovary cycle disturbances	Case of unphysiological cycle within day 51 until 305 after calving	26,884

Abbreviation and name of the individual traits, definition according to VIT [11] as well as number of animals per trait (No.). Compared with the literature, for some traits synonyms are used [16, 28]. For example, “paternal” and “indirect” are synonymously utilised for “direct” and “maternal”, for reason of clarity, we used the terms as named by VIT, also later compared with other studies, in case of matching trait definitions

### Genotyping data, quality control

About 96% of the animals were imputed to 50 K level from various versions of the EuroGenomics low-density (LD) chips (Eurogenomics, Amsterdam, NL), while the rest was genotyped with various versions and quality of the Illumina 50 K chips (Illumina Inc., San Diego, CA) and EuroGenomics medium-density (MD) chips (Eurogenomics, Amsterdam, NL). This imputation procedure is described in Segelke et al. [29]. After lifting to reference genome assembly ARS.UCD1.2, 45,613 SNPs of 252,285 cows were imputed to sequence level in two steps. Firstly, animals were imputed to a high-density reference panel (∼ 777 K), and from that to the whole genome sequence level using the Run9 reference panel of the 1000 Bull Genomes consortium [19]. Imputation was performed using Beagle 5.2 [30]. Afterwards, data were quality filtered using the dosage R squared parameter (DR^2^ > 0.75) [31] and a minor allele frequency cutoff of 1%. A detailed description of the workflow and filtering criteria can be found in Križanac et al. [32]. The final dataset consisted of 17.2 million SNPs. Given the entirely female and large sample size, for subsequent analyses we included the X-chromosomal information to facilitate a more comprehensive examination. This aspect was highlighted in recent studies as the X chromosome may be a potential and important source of information (e.g [33, 34]).

### Pedigree based analyses

For the estimation of variance components, Bayesian uni- and bivariate animal models using Markov chain Monte Carlo sampling techniques with a Gibbs sampler as implemented in the R-package MCMCglmm [35] were used. The chain length was 25,000 iterations and the burn-in 5,000 iterations. Traits with a binary coding (namely **MET**, **ZYS**, **GME**, **NGV**, **NR56**, **NR90**, **NRh**, **SBm**, and **CEm** recoded as binary) were analysed with a generalized linear mixed model. The underlying latent variable 𝑙𝑖 with *i* as the liability for the *i*-th animal is connected to the binary observation $\:{y}_{i}$ with a probit link function in the model. Choosing a probit over a logit link function is the more accurate choice for an animal model [36].

$$\:{y}_{i\:}\sim\:B\left({probit}^{-1}\left({l}_{i}\right)\right)$$

$$\:l\:=\:\:\mu\:\:+\:X\beta\:\:+\:Za\:+\:Whys\:+\:e\:\:\:$$

The model was defined with $\:{l}_{i}$ as the vector of liabilities for each individual and 𝜇 as the mean liability, 𝛽 being the vector of fixed effects for age at first calving (EKA), 𝑎 the random effects for the additive genetic effect, ℎ𝑦𝑠 a multicode effect set up of heard, year and season of the individual cow and 𝑒 the residuum with a fixed residual variance of 1. 𝑎 and ℎ𝑦𝑠 are said to be normally distributed with $\:a\:\sim\:N(0,A{\sigma\:}_{a}^{2})$ and $\:hys\:\sim\:N(0,I{\sigma\:}_{hys}^{2})$, with *A* as the pedigree-based relationship matrix and *I* indicating the identity matrix. The prior for both effects was set to follow a $\:{\chi\:}^{2}$ distribution with one degree of freedom. Heritabilities for traits with binary coding were calculated with the following formula.

$$\:{h}^{2}=\:\frac{{\sigma\:}_{a}^{2}}{{\sigma\:}_{a}^{2}+\:{\sigma\:}_{hys}^{2}+\:{\sigma\:}_{e}^{2}+1}$$

For binary traits, residual variance was fixed to 1. The remaining traits, namely **CFc**, **DOc**, **FSc**, **FSh**, and **CEm** as linear, follow a normal distribution and were therefore analysed using the same model, but without the probit link. Prior assumptions are weak with an uninformative prior following $\:{\sigma\:}_{a}^{2}\:\sim\:inv-gamma\left(\text{0.01,0.01}\right)$ and $\:{\sigma\:}_{hys}^{2}\:\sim\:inv-gamma\left(\text{0.01,0.01}\right)$. $\:{y}_{i}$ is the vector of observations. Next to EKA also days in milk (DIM) was chosen as fixed effect.

$$\:y\:=\:\:\mu\:\:+\:X\beta\:\:+\:Za\:+\:Whys\:+\:e\:\:\:$$

Trait heritability for these models was calculated in the following manner

$$\:{h}^{2}=\:\frac{{\sigma\:}_{a}^{2}}{{\sigma\:}_{a}^{2}+\:{\sigma\:}_{hys}^{2}+\:{\sigma\:}_{e}^{2}}$$

Calving ease maternal was analysed in two ways for variance components. In a first run **CEm** was used as a linear trait and all four factor levels were maintained. In a second approach the levels were merged to achieve a recoded binary trait, where “easy” and “normal” as well as “heavy” and “with vet / caesarean” were taken together, respectively.

### Genome-wide association analysis (GWAS)

For genome-wide association analysis, a mixed linear model approach (MLMA) as implemented in the software tool for genome-wide complex trait analysis (GCTA) version 1.93.2 beta was applied [24, 37] using the following model:

$$\:y=a+bx+g+e$$

where *y* is the vector of DRPs, *a* is the mean term, *b* is the additive affect (fixed effect) of the candidate SNP to be tested for association, *x* is the SNP genotype indicator variable coded as 0, 1 or 2, *g* is the polygenic effect captured by the genetic relationship matrix (GRM) and *e* is the residual. The GRM was calculated between pairs of individuals from the set of SNPs used on chip level before imputation including 44,144 SNP-markers on the autosomes, using the approach of Yang et al. [38]. The majority of GWAS in dairy cattle only relies on autosomes [14, 39]. However, we included the X chromosome as a potential source of information for fertility and reproduction traits due to the previously disclosed relationship between phenotypic expression and sex chromosomes in the context of reproductive performance [33, 40]. In addition, it has been identified that X-linked genes have an significant influence on various complex traits in dairy cattle, including reproduction of Holstein dairy cattle [34]. Threshold for significance of the GWAS statistic was Bonferroni corrected on genome-wide level to account for multiple testing of SNPs included in the MLMA approach with a level of 0.05 ([(0.05/17,222,496), p = 2.903 * 10^*− 9*^]). The R package ggplot2 [41] was used in R version 4.2.0 [42] to generate the Manhattan plots for graphical representation.

### Variant effect prediction

Ensembl Variant Effect Predictor (VEP) [43] was used to downstream analyse genome-wide significantly associated SNPs. All genes taken into account were known genes confirmed by an approved symbol of the gene nomenclature [44]. A gene was considered significantly associated with a trait if at least one SNP in proximity reached the Bonferroni threshold ([(0.05/17,222,496), p = 2.903 * 10^*− 9*^]). For proximity, a window of 10,000 base pairs (bp) downstream and upstream of the variant, according to genome assembly ARS-UCD 1.2, was considered.

### Downstream analyses

Enrichment analyses were conducted using the R packages org.Bt.eg.db [45], clusterProfiler [46] and DOSE [47] using default settings. SNPs with MLMA results below p < 1 * 10^*− 4*^ were included. To assess the over-representation of genes belonging to particular pathways, we used the Kyoto Encyclopedia of Genes and Genomes (KEGG) [48] database for enrichment. The tool g:Profiler [49] was used with default settings to determine the molecular function of gene products and cellular components specific for the findings on the X chromosome only. The R package VennDiagram [50] was used to generate Venn diagrams showing shared candidate genes.

## Results

### Heritabilities

Observations for events and their incidences are shown in Table 2.

**Table 2:** Overview about analysed traits, number of observations per trait and affiliated incidences

Trait	Abbreviation	Observations	Incidence (in %)
Endometritis	MET	3422	9.92
Ovary cycle disturbances	ZYS	5228	15.64
Disease of the uterus	GME	5394	15.15
Retained placenta	NGV	1472	4.27
Non-return rate 56 days cow	NR56	17,860	51.77
Non-return rate 56 days heifer	NRh	10,965	31.79
Non-return rate 90 days cow	NR90	19,962	57.87
Stillbirth	SBm	2360	6.84
Calving ease “easy”	CEm	27,233	78.94
Calving ease “normal”	CEm	6014	17.43
Calving ease “heavy”	CEm	1218	3.53
Calving ease “with vet / caesarean”	CEm	32	0.09

Based on these binary traits and the recoding of **CEm**, the results for genetic parameter estimations are shown in Table 3. The heritability (h^2^) for binary traits is based on liability scale with a range of 0.014 (**MET**) to 0.211 (**SBm**). For **MET** the lower 95% quantile of both heritability estimates were close to zero.

**Table 3:** Genetic parameters from variance components estimation

Abbreviation	$\:{\sigma\:}_{a}^{2}$	$\:HDP\:{\sigma\:}_{a}^{2}$	$\:{\sigma\:}_{hys}^{2}$	$\:{\sigma\:}_{e}^{2}$	$\:{h}^{2}$	$\:HDP\:{h}^{2}$
MET	0.055	< 0.001–0.125	1.891	1	0.014	< 0.001–0.031
ZYS	0.134	0.078–0.207	3.784	1	0.023	0.013–0.034
GME	0.116	0.037–0.192	1.860	1	0.029	0.011–0.048
NGV	0.393	0.231–0.581	0.969	1	0.116	0.075–0.164
NR56	0.062	0.024–0.101	0.281	1	0.026	0.010–0.042
NRh	0.067	0.024–0.010	0.1904	1	0.029	0.013–0.046
NR90	0.087	0.042–0.132	0.380	1	0.035	0.018–0.053
CEm^1^	0.195	0.073–0.353	0.614	1	0.069	0.029–0.119
SBm	0.796	0.555–1.057	0.123	1	0.211	0.211–0.335
CFc	25.080	16.810–31.720	102.200	489.300	0.041	0.027–0.051
FSc	150.800	109.100–191.500	184.100	3244.000	0.042	0.031–0.054
FSh	84.190	58.740–108.600	56.430	1397.000	0.054	0.038–0.070
CEm^2^	0.012	0.007–0.017	0.034	0.216	0.045	0.027–0.064
DOc	225.400	179.600–270.100	240.800	3430.000	0.057	0.046–0.069

Additive genetic ($\:{\sigma\:}_{a}^{2}$), multicode herd-year-season ($\:{\sigma\:}_{hys}^{2}$). residual variance ($\:{\sigma\:}_{e}^{2}$) and heritability ($\:{h}^{2}$). In case of $\:{\sigma\:}_{a}^{2}$ and $\:{h}^{2}$ in addition the highest posterior density (HDP) interval is given ($\:HDP\:{\sigma\:}_{a}^{2}$ and $\:{HDP\:h}^{2})$. For calving to first insemination (**CFc**) and first to successful insemination (**FSc/FSh**) a timespan in days is given instead of binary coding. CEm^1^ calving ease maternal, analysed as binary trait, recoded like described in pedigree based analyses section. CEm^2^ is calving ease maternal, analysed as linear trait with all factor levels, also described in the in pedigree based analyses section. Abbreviations of traits are explained in detail in Tables 1 and 2

### GWAS results

We obtained over 2700 genome-wide SNPs significantly associated with 9 out of the 13 traits on various chromosomes as shown in Table 4. The significantly associated SNPs were located on *Bos taurus* (BTA) chromosome 6 (BTA6), BTA18 and the X chromosome (BTAX). Four different traits were associated with regions on BTA6, whereas the highest number of significantly associated SNPs embracing the broadest regions was found on BTAX.

### Chromosome 6

**Fig. 1:**
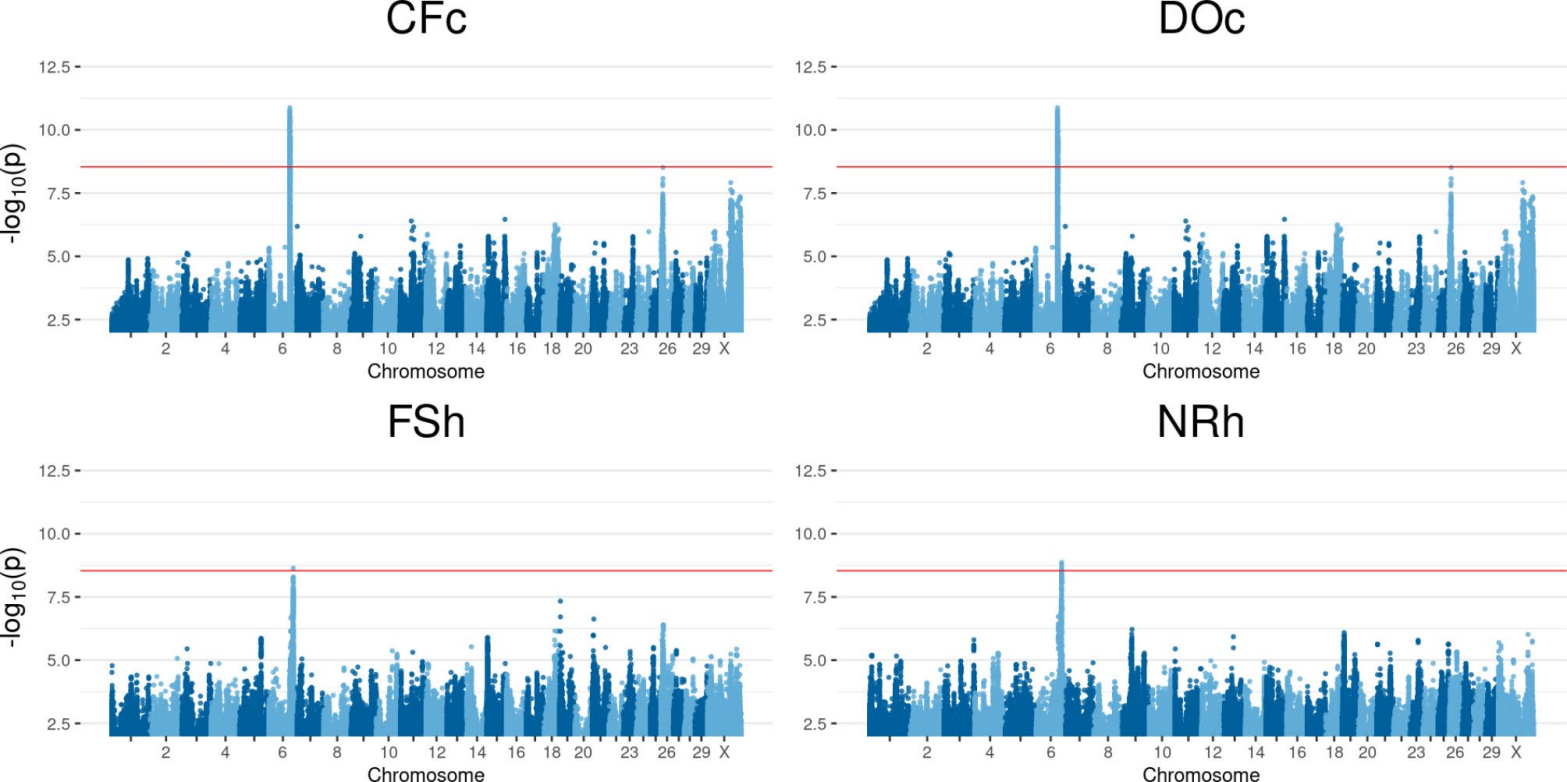
Manhattan plots of QTL mapping results for significant traits on chromosome 6. Calving to first insemination (**CFc**), days open (**DOc**), first to successful insemination (**FSh**) and non-return rate heifers (**NRh**). Negative decadic logarithm of *p*-value of each SNP is shown on the y-axes, on x-axes the 29 autosomes and X chromosome is shown. The red line represents the significance threshold on genome-wide level p = 2.903 * 10^*− 9*^

In total, 952 significant SNPs and six associated genes were identified for first to successful insemination heifer (**FSh**), non-return rate heifer (**NRh**), calving to first insemination cow (**CFc**) and days open cow (**DOc**) on BTA6. The results for these traits are shown in Fig. 1 and the dedicated number of SNPs per trait listed in Table 4. For **FSh** a significant SNP in 211 bp distance to *PTPN13* reached genome-wide significance level. Four tested SNPs appeared significant for **NRh** between 102,025,927 bp and 102,081,667 bp and directly associated with *AFF1* and *KLHL8* (two hits for each gene). Within a window from 86,745,798 bp and 87,358,291 bp, a total of 947 SNPs showed up significant for **CFc** and **DOc**. Almost a fourth, approximately 220 SNPs, were detected for both traits and were assigned to *SLC4A4*, *GC* and *NPFFR2*.

### Chromosome 18

**Fig. 2:**
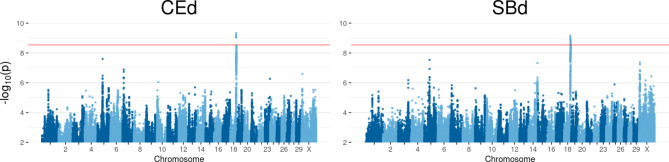
Manhattan plots of QTL mapping results for significant traits on chromosome 18. Calving ease direct (**CEd**) and stillbirth direct (**SBd**). Negative decadic logarithm of *p*-value of each SNP is shown on the y-axes, on x-axes the 29 autosomes and X chromosome is shown. The red line represents the significance threshold on genome-wide level p = 2.903 * 10^*− 9*^

Analyses of **CEd** and **SBd** led to significant peaks on BTA18 for both traits, shown in Fig. 2. For **CEd** eight SNPs reached Bonferroni threshold. The SNPs found were between 57,055,186 bp and 57,062,793 bp and displayed an association with *CTU1*. Regarding **SBd**, the same *CTU1* associated SNPs could be found. In addition, significant associated SNPs were identified in a distal region on BTA18 between 59,506,758 bp and 60,085,291 bp.

### X Chromosome

**Fig. 3:**
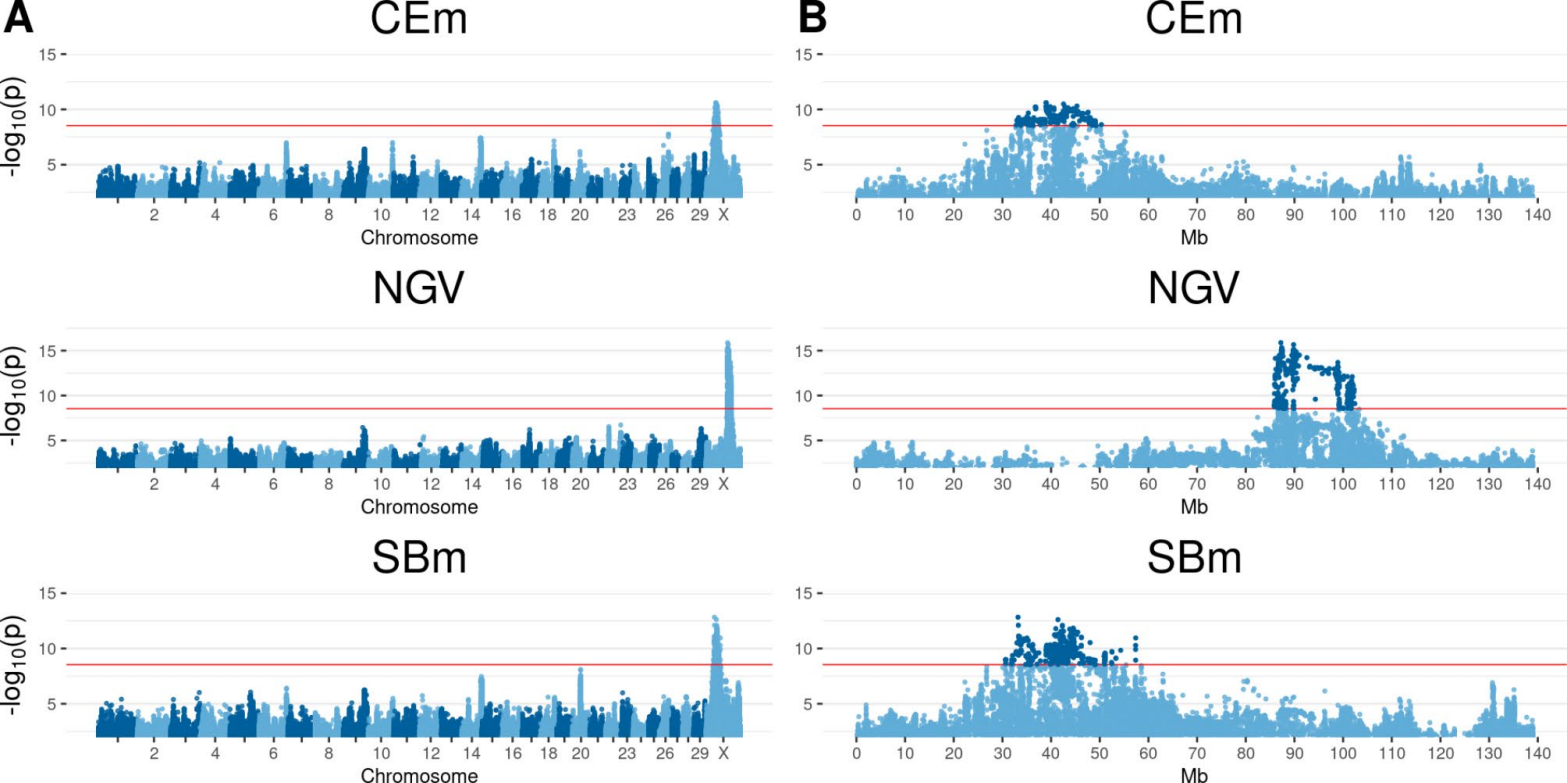
Manhattan plots of QTL mapping results for significant traits on chromosome X. Calving ease maternal (**CEm**), retained placenta (**NGV**), and stillbirth maternal (**SBm**). (**A**) Genome-wide results, negative decadic logarithm of *p*-value of each SNP is shown on the y-axes, on x-axes the 29 autosomes and X chromosome is shown. The red line represents the significance threshold on genome-wide level p = 2.903 * 10^*− 9*^. (**B**) The detailed presentation of BTAX per trait, including the significantly associated region, Megabase pairs (Mb) are shown on the x-axes. The SNPs that exceed the significance threshold are highlighted in dark blue

For three traits (**CEm**, **SBm**, **NGV**) several genome-wide significant SNPs were detected on BTAX, including a various number of associated genes (Fig. 3 and Additional file 1). For **SBm**, a region between 30,670,566 and 57,379,944 bp was found to be significantly affected by 1233 SNPs. A set of 35 different genes was found to be associated with the SNPs. For **CEm**, a region between 32,856,421 bp and 50,341,339 bp, including 328 SNPs with a total of 26 announced genes, were identified. An overlap of 22 genes between 32,856,421 bp and 45,950,746 bp shared a number of common significant SNPs for **CEm** and **SBm**. Between 85,905,388 bp and 102,397,780 bp, a total of 838 significant SNPs were identified for **NGV**, along with 45 different genes described within this region.

**Table 4:** Traits with genome-wide significantly associated SNPs and their linked chromosomes

	CEd	CEm	CFc	DOc	FSh	NGV	NRh	SBd	SBm
BTA6			X	X	X		X		
BTA18	X							X	
BTAX		X				X			X
λ	1.042	1.042	1.022	1.064	1.043	1.017	1.058	1.055	1.087
SNPs^1^	8	328	281	666	1	838	4	20	1233
Genes^2^	1	26	3	3	1	45	2	1	35

The genomic inflation factor (λ) for each tested trait and affiliated mixed-linear model, as well the number of genome-wide significant SNPs per trait ^(1)^. Moreover, genes located in a 10,000 bp (bp) window down- and upstream the associated SNPs ^(2)^

### Gene enrichment

**Fig. 4:**
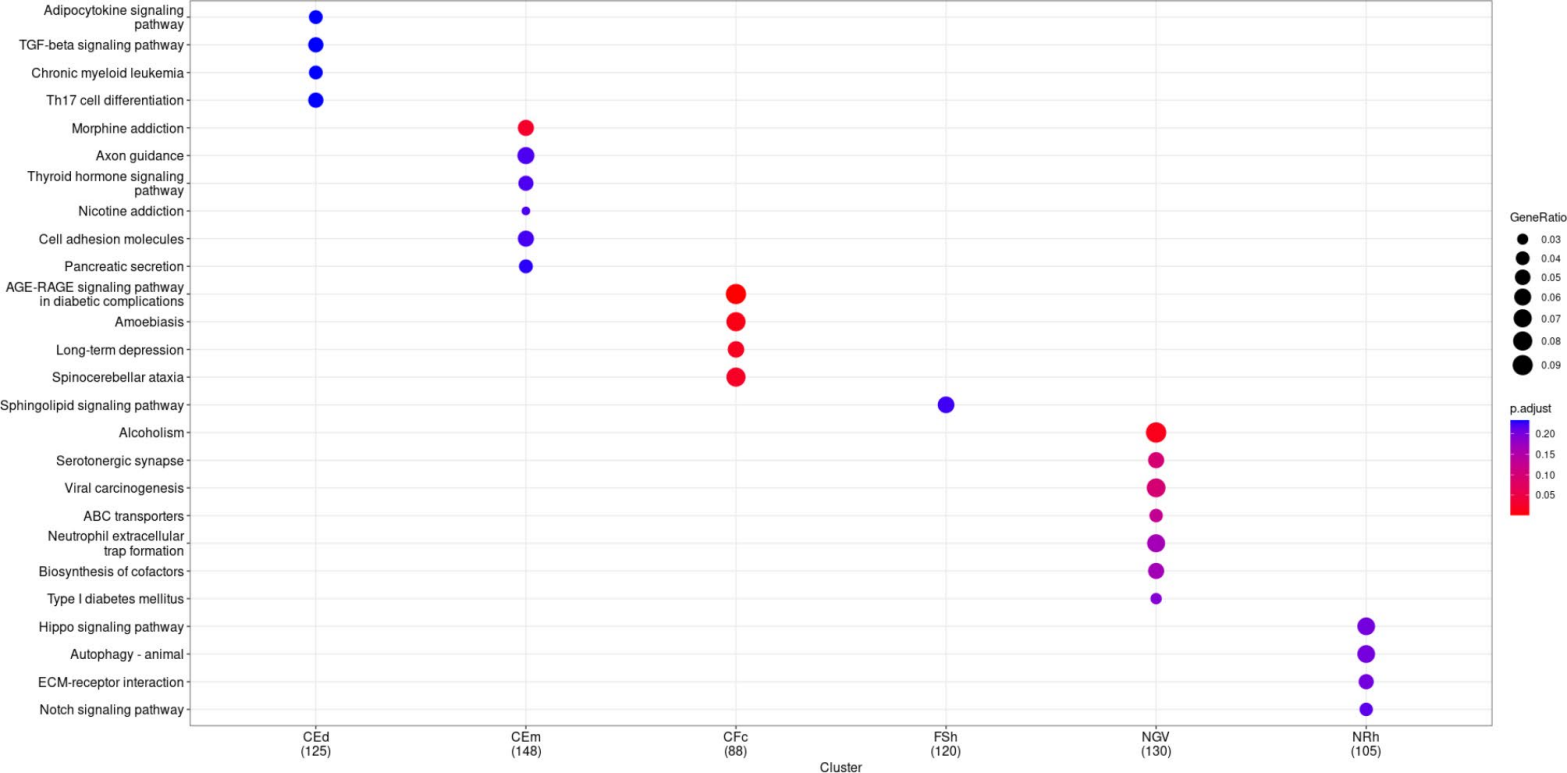
Functional enrichment dot plot. Calving ease direct (**CEd**), calving ease maternal (**CEm**), calving to first insemination (**CFc**), first to successful insemination heifer (**FSh**), retained placenta (**NGV**) and non-return rate heifers (**NRh**). The result for over-represented genes in 26 different pathways (y-axis) and the corresponding traits (x-axis). The thickness of dots represents GeneRatio and the color represents the associated adjusted *p*-value

The functional enrichment analysis was performed for all trait related genes identified in GWAS. To evaluate the list of reasonable genes, we related the genes identified by the genome-wide significant associated SNPs in GWAS to those identified with the SNPs showing a *p*-value p < 1 * 10^*− 4*^. No significant results were obtained for **DOc**, **SBd**, and **SBm**. For the other six traits, we identified over-represented genes in 26 different pathways using the KEGG database. The results were specific to each trait, with no overlap between them. Figure 4 shows a common dot plot used for visualising the functional enrichment results. For BTAX, an additional enrichment approach was conducted using the g:Profiler tool [49]. The results of this approach are presented directly in the discussion and summarized in context. In this context, 33 distinct genes out of 83 genome-wide significant identified on BTAX (detailed summary in Additional file 1) could finally be evaluated in the context of the in-depth enrichment analysis.

## Discussion

This study analysed a sample of 34,497 primiparous German Holstein cows to estimate genetic parameters for reproduction traits based on first lactation records using pedigree-based analyses. Additionally, DRPs were used to perform GWAS on imputed WGS data and downstream analyses. This enabled the estimation of variance components for these functional traits and identification of genome-wide significant SNPs, indicating potential candidate genes. Genetic parameters from variance component estimation showed results in line with previous studies for cattle [4, 9, 11], for example h² = 0.041 for **CFc** [9], and therefore the suitability of our subset for the conducted analyses. Due to the comprehensive number of animals included in this analysis, even for the low heritable functional traits (e.g. **MET**), reliable results escorted by tight HPD intervals were obtained. Like outlined by Berry et al. [4] for reproductive performance, various factors can affect the variance component estimation, also resulting in a population specific estimation. Nevertheless, there are limitations arising from the available data here, as only reproductive performance of the first lactation was taken into account, regardless of for how long each animal stayed in the population, although this is highly linked to reproductive performance and vice versa. DRPs provide an alternative method for handling traits with raw phenotypes, which are binary coded, while also allowing for the consideration of multiple observations within and between lactations. This increased the variance within traits to more accurately identify associated SNPs or regions [51]. The primary advantage of DRPs is their greater informativeness compared to raw observations of own performance, as they are estimated using the full reference population. An additional weighting could have been added, such as effective daughter contributions [52], but due to the selection of the cohort and therefore pre-correction of similar information quality origin from the same reference population, additional weighting was not applied. Striking signals were found on two autosomes (BTA6, BTA18) and BTAX. Testing more than 17.2 million SNPs in this large cohort led to an increased capability to identify associations for the tested functional traits. Furthermore, the inclusion of BTAX allowed us to identify two major regions including several genes in context of reproductive performance and revealed a new potential information source.

### Chromosome 6

A significantly associated SNP was found on BTA6 at 101,529,627 bp located with *PTPN13* (protein tyrosine phosphatase non-receptor type 13) for **FSh** in a 2118 bp distance. Kolbehdari et al. [53] detected this gene as chromosome-wide significant, affecting direct calving ease. The *PTPN13* encoded protein belongs to the protein tyrosine phosphatase (PTP) family, which are signalling molecules involved in various cellular processes such as cell growth, differentiation, and mitotic cycle processes [54]. In contrast, no peak or genome-wide significant associated SNP was detected for **FSc**. It could be hypothesized that *PTPN13* influences heifers and cows to different extents. Heifers undergo their own growth and development, in addition to potential embryonic development after successful insemination [55]. This could lead to bias according to the heifer’s own demands for development and an overall increase in cell growth and differentiation, which could explain the difference between **FSh** compared to **FSc** in terms of association results. In humans, *PTPN13* is discussed as a potential tumour suppressor that regulates cell growth in various tissues, resulting in better outcomes for those affected [56, 57]. This suggests that *PTPN13* may have two potential points of interaction. Firstly, the development of heifers requires resources for growth and tissue differentiation, unlike adult cows where requirements are no longer divided into growth and maintenance. During placentation and gestation, there is a physiological process of tissue development and remodelling [49].

The significantly associated SNPs for **NRh** were identified between 102,025,927 and 102,081,667 bp and were found to be locally close to *AFF1* (ALF transcription elongation factor 1) and for *KLHL8* (Kelch like family member 8) SNPs were within and close to the gene. *AFF1* has already been suggested in the literature to be associated with conception rate in dairy cows [58] and as important transcription factor in the molecular regulation of puberty in beef cattle [59]. Furthermore, *AFF1* was reported to influence daughter pregnancy rate, cow and heifer conception rate including a large dominance effect for heifer conception rate [39]. Specific gonadal studies identified *AFF1* expression in the ovary, epididymis, and testis of mice [60]. Our results with an association between **NRh** and *KLHL8* are in agreement with the literature. *KLHL8* was previously proposed as a potential candidate gene for **NRh** and involved in oogenesis [61]. In a study by Koh et al. [62], *KLHL8* was detected in plasma of low-fertility heifers functionally assigned to cellular and metabolic processes. Exosomes of high and low fertility heifers were isolated from plasma, processed and afterwards analysed by mass spectrometry. The *KLHL8* product, Kelch-like protein 8, was one of two unique proteins only present in plasma of low fertility heifers. Beside this, Koh et al. [62] further showed a katalytic activity of both unique proteins.

A region with overlapping association signals was found on BTA6 for **CFc** and **DOc**, as **DOc** is dependent on **CFc** and **FSc**. Therefore, it is reasonable to assume that a shared genomic region affects both traits. The region containing SNPs that are significantly associated with both traits is situated between 86,745,798 bp and 87,358,291 bp. It has been previously described that *SLC4A4* (solute carrier family 4 member 4) is associated with milk production and clinical mastitis [63, 64], as well as with somatic cell score [39]. *SLC4A4* is involved in the regulation of bicarbonate secretion and absorption, encoding a sodium-bicarbonat-cotransporter [54]. For neonatal Holstein calves, the role of *SLC4A4* inside metabolic pathways and intracellular pH control in ruminal epithelium tissue was assessed using gene expression analysis [65]. It is possible that particular characteristics of the endometrium affect the implantation process.

*GC* (GC vitamin D binding protein) is involved in vitamin D metabolism together with transport and was already described in context of milk fever [66], mastitis resistance [63, 64], body condition score and calving interval [63] in dairy cattle. In humans, several associations between vitamin D-binding Protein and reproductive health were described [67]. It is known that pregnancy increases the demand of vitamin D throughout the time of pregnancy and lactation and in case of deficiency, the metabolic system is incapable to fulfil the requirement neither of the mother, nor the developing foetus [68]. Vitamin D is involved in different processes according to reproduction and production traits, underlined through the several traits found in literature. This indicates a possible direct effect on reproductive performance due to a limited amount of vitamin D for the developing foetus. On the other hand, there may be an indirect effect through an increased demand for milk yield during early lactation, which could lead to decreased reproductive performance, resulting in extended time for **CFc** and **DOc**.

*NPFFR2* (neuropeptide FF receptor 2) is a G protein-coupled receptor for neuropeptide FF, which is involved in modulating the opioid system and regulating cardiovascular and neuroendocrinological function [69]. Bonini et al. [70] described an upregulated mRNA expression of *NPFFR2* in the human placenta. In addition, there is a known physiological interaction between endogenous opioids and gonadotropin secretion in various mammalian species [71, 72]. It is therefore reasonable to assume that any deviation from the hormonal control cycle may result in a disruption that makes it more difficult to achieve pregnancy, thereby increasing the time between calving and successful insemination.

### Chromosome 18

On BTA18, SNPs significantly associated with **CEd** and **SBd** were located in a region between 57,005,186–60,085,251 bp, which encompasses the *CTU1* (cytosolic thiouridylase subunit 1) gene. *CTU1* is involved in the sulphur relay system in humans [73] along with tRNA modifications of the uridine at position 34. This modification occurs in an interplay with the estrogen receptor α [74]. Abo-Ismail et al. [14] were able to detect a region on BTA18 between 56.9 and 59.9 Mb, including *CTU1*, associated with calving performance and rump traits. The potential interplay with the estrogen receptor appears to be a plausible physiological reason for the association between **CEd** and **SBd** and restrictions in light calving. The association of the rump trait may also be linked to the ability of light calving or even an increased likelihood of heavy births, as shown by Cue et al. [75].

### X Chromosome

**Fig. 5:**
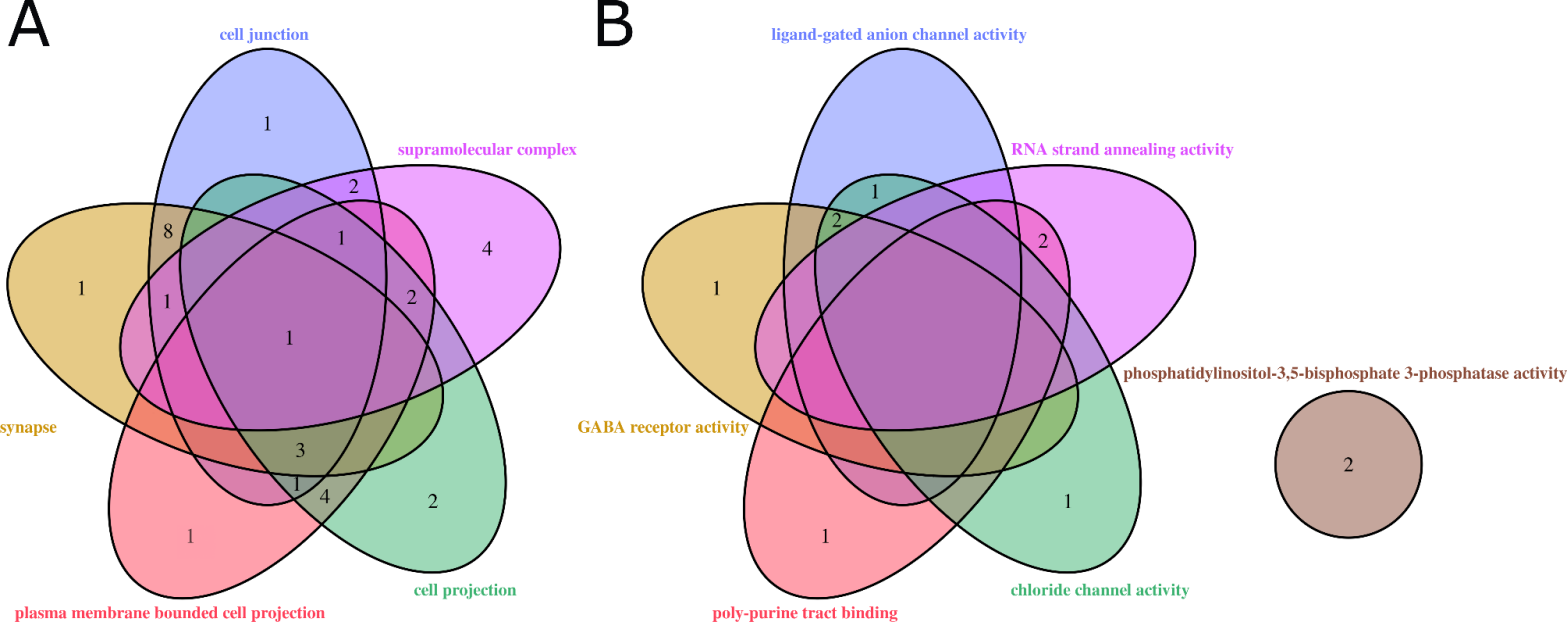
Venn diagram of BTAX associated genes. Gene associations according to the g:profiler results. Overlapping shapes represent interception between different parts for the same gene. (**A**) Associated cellular components for identified genes. (**B**) Associated molecular functions for identified genes

To gain a better understanding of the significant SNPs found for **CEm**, **NGV** and **SBm** on BTAX, we utilised the g:Profiler tool [49]. By using the g:GOSt option, we conducted an enrichment analysis for the identified genes. Since there is a lack of evidence-based biological processes that are known to be associated with the gene products, we outline the molecular functions of the gene products. In addition, we outline the cellular components to describe the physical location of a gene product in the cell. Default settings were used. Additionally, we considered the dependency of multiple testing due to the overlap of functional terms, as described in Reimand et al. [76]. Figure 5 presents the results for molecular function and cellular components as Venn diagrams.

A total of 33 distinct genes were identified, 11 of which were identified for molecular function, 30 for cellular component, and seven were shared between both categories (*FMR1*, *MTM1*, *GABRA3*, *PABPC5*, *GLRA4*, *CLCN5*, *DDX3X*). The cellular component category had the highest variation of genes, ranging from 11 different genes for ‘supramolecular complex’ to 18 genes for ‘cell junction’. To refine our results, we focused on genes that were most frequently affected in function or components for each trait tested that was associated with BTAX. The identified gene was then considered as the most promising gene.

Starting with the cellular components (as shown in Fig. 5A), the gene *FMR1* (Fragile X messenger ribonucleoprotein 1) was found to interact with all five available components. *FMR1* is located in the region between 30,624,825 and 30,664,682 bp harbouring several SNPs significantly associated with the trait **SBm**. Additionally, *FMR1* is involved in two molecular functions: ‘RNA strand annealing’ and ‘poly-purine tract binding’. The *FMR1* gene is well-described in humans in the context of disturbed fertility in women and the associated Fragile X Syndrome (FXS) [77]. This effect is associated with a dynamic mutation that increases the number of CGG triplet repeats across generations. A variation in methylation and the number of CGG repeats occurs within the untranslated region of the first exon [78, 79]. This circumstance leads to an ovarian dysfunction and is physiologically associated with an increased level of FSH, premature ovarian failure or an earlier menopause before the age of 40 years [80, 81]. Mihm et al. [82] identified a decline in FSH levels as a crucial factor in the selection process of the dominant follicle in cattle. These physiological interactions are consistent with the characteristics of **CEm**, **NGV** and **SBm**. The changes in FSH levels and CGG triplet numbers may affect birth-related observations. The extent of this variation seems to be logically derived from the stages that are influenced, both in terms of time and intensity. These stages cover severe births, stillbirths and postnatal behaviour.

The gene *GABRA3* (Gamma-aminobutyric acid type A receptor subunit alpha 3), which encodes for the gamma-aminobutyric acid type A receptor subunit alpha 3, showed a cluster between four of the five components, except for ‘supramolecular complex’, including significant associated SNPs for **CEm** and **SBm**. This gene is located at 34.602 Mb up to 34.842 Mb on BTAX, bordered by a region including further genes matching gamma-aminobutyric acid type A receptor subunits (*GABRE*, *GABRQ*). Both of these genes also showed a significant association of SNPs with **CEm** and **SBm**. *GABRA3* encodes the alpha 3 subunit in GABAA receptors, which is part of a receptor complex that exhibits functional diversity depending on subunit composition [83]. The alpha 3 subunit is associated with both anxiogenesis and anxiolysis, as described by Atack et al. [84] and Dias et al. [85]. Additionally, Rudolph et al. [86] stated that the muscle relaxant activity of diazepam is mediated by this subunit in mice. In humans, *GABRA3* is associated with fetal brain development and is considered a candidate gene for Rett syndrome, a neurodevelopmental disorder that primarily affects females [87]. GABAA receptors are critical binding structures for allopregnanolone, which acts as a potent allosteric modulator for these receptors. Allopregnanolone concentrations vary during pregnancy and play an important role in protecting pregnancy and birth outcomes in various mammalian species, including sheep and humans [88]. Sheep and cattle, both ruminants, have similar placentation ratios. Therefore, it is reasonable to compare the effect observed in the foetal brain of sheep and lambs with that of cows and calves in terms of hormonal circulation. In sheep, the level of allopregnanolone increases during late gestation, reaching a maximum near term, and then decreases after birth [88]. An imbalance in this regulation, due to a lack of *GABRA3*, could explain the problems around birth, compared to cattle and both traits **CEm** and **SBm**.

*SYN1* (Synapsin I) was identified for **NGV** and shared the same four cellular components as *GABRA3* described above. *SYN1* is located more distally on BTAX between 85.929 Mb and 85.992 Mb and is a member of a gene family that encodes for neuronal phosphoproteins [54]. Synapsins play an essential role in regulating vesicles, especially in accelerating synaptic vesicle traffic through repetitive stimulation [89]. In the context of neurological disorders, many diseases are associated with synapsins. Their expression patterns are described in the literature [90]. Synapsin 1, in particular, has been highlighted as an important mediator for glucocorticoids [91] and has been detected as a member of hormonal adjustment in GnRH and LH release [92] in the field of reproductive physiology. In dairy cattle, GnRH treatment has been shown to have a positive effect on reducing the number of services per conception and shortening the days open in cases of **NGV** [93]. However, GnRH is generally proposed as a mediator to improve fertility in low or moderate fertility cows, especially heifers [94]. It may be difficult to separate the general hormonal effect from the clinical persistence of **NGV**. A decreased neutrophil function and recruitment has been linked to causing **NGV** in dairy cattle [95]. Neutrophils are involved in the immune response to treatments such as infections or injuries. It seems reasonable to expect an effect in the case of **NGV** when the decreased function in neutrophils is challenged by an infection reaction.

For molecular functions (refer to Fig. 5B), fewer genes are related compared to the results for cellular components, and there is a lower proportion of shared elements. The previously discussed *GABRE3* accounts for the majority of functions, including ‘ligand-gated anion channel activity’, ‘GABA-receptor activity’ and ‘poly-purine tract binding’. Based on the intersection between molecular function and cellular component, the *PABPC5* (Poly(A) binding protein cytoplasmic 5) gene, appears to be the most likely candidate to affect reproductive performance issues such as **SBm** and **CEm** in cattle.

*PABPC5* was identified for **SBm** as well as **CEm**. It belongs to the cytosolic poly(A) binding protein family and is involved in protein binding at the 3’ end of the poly(A) tail [96]. Its location on BTAX is between 39.337 Mb and 39.339 Mb. An involvement in mitochondrial metabolism and apoptosis is described [97], and an association between premature ovarian failure in ovarian diseases as well as ovarian cancer is linked to *PABPC5* in humans [98]. Furthermore, this is underlined functionally by *PABPC5* expression in testis and ovarian tissue [98] and the known involvement of poly(A)-binding proteins in germ cell development [99].

The enrichment of the identified genes on BTAX indicates the contribution of this gonosome to reproductive performance. Two regions have shown significant associations with different calving and fertility traits. The relationship between hormones and tissues of the dam and offspring during gestation is a complex and physiologically well balanced interplay. Therefore, the number of accounting genes seems reasonable. Several studies have been conducted to investigate the multifactorial problems affecting reproductive performance in dairy cattle [28, 100, 101]. The genes presented in this section, as determined by enrichment analysis, have a direct influence on reproductive performance (e.g. *FMR1*) or are linked to pregnancy-dependent physiological processes (e.g. *SYN1*). However, the use of BTAX for GWAS is still increasing. Methods to improve the joint inclusion of autosomes and gonosomes are currently under development [34, 102]. KEGG pathway enrichment analyses also revealed a high proportion of signalling pathways (7 out of 26), distributed across almost all traits. This is consistent with the previously presented genes, such as *PTPN13* [54] or *SLC4A4* [65], which were also identified in the context of signalling pathways. It is important to note that the associated adjusted *p*-values should be viewed with caution, as they may make it more difficult to draw strong conclusions from the results of the KEGG enrichment analysis. Only three traits achieved an adjusted *p*-value below 0.05. The difficulty in quantification may be attributed to the low proportion of previously identified genome-wide significantly associated genes. However, the corresponding lambda values of the GWAS statistics suggest that the detected number of SNPs and their associated genes are unlikely to have been overestimated. It is known that the detection of associated markers in functional traits is challenging [9], which may limit potential downstream analyses. However, the results from g:Profiler suggested a promising approach for narrowing down and enriching downstream analyses as a follow-up to the obtained GWAS results.

## Conclusion

We identified candidate genes for fertility in dairy cattle. More than 2700 genome-wide significantly associated SNPs were detected representing more than 90 different genes. Major association signals on BTA6 and BTA18 are in line with previous research, while our WGS based approach, in conjunction with downstream analyses, allowed for the identification of putative candidate genes on BTAX. Some of the genes, such *FMR1* and *PABPC5* have been directly related with reproductive disturbances in humans, mice, or sheep. Considering the interplay between reproduction and performance in dairy cattle, the relevance of BTAX appears to be evident. Thus, the analyses can help to better explain the genomic architecture for reproduction traits.

## Electronic supplementary material

Below is the link to the electronic supplementary material.

Additional file 1: Significant associated genes on BTAX: Result table for identified genes on BTAX and corresponding number of SNPs per trait, as well as position on the chromosome. Included traits are calving ease maternal (**CEm**), retained placenta (**NGV**) and stillbirth maternal (**SBm**)

Additional file 2: Genome-wide significant SNPs per trait: Results of GWAS summary statistics for all genome-wide significant SNPs identified per analysed trait. Results are separate for each trait, namely, calving ease direct and maternal (**CEd/CEm**), calving to first insemination (**CFc**), days open (**DOc**), first to successful insemination heifer (**FSh**), retained placenta (**NGV**), non-return rate 56 heifer (**NRh**) and stillbirth direct and maternal (**SBd/SBm**)

## Data Availability

The SNP chip genotype data and phenotypes cannot be shared publicly, as they are property of the German Holstein breeding organisations organised in the umbrella federation BRS (Bundesverband Rind und Schwein e.V., Bonn, Germany), deregressed proofs are computed by the national computing center VIT (Vereinigte Informationssysteme Tierhaltung w.V., Verden) in Germany. Summary statistics can be provided by the senior author JT (jens.tetens@uni-goettingen.de) upon reasonable request.
